# The “DOC” screen: Feasible and valid screening for depression, Obstructive Sleep Apnea (OSA) and cognitive impairment in stroke prevention clinics

**DOI:** 10.1371/journal.pone.0174451

**Published:** 2017-04-04

**Authors:** Richard H. Swartz, Megan L. Cayley, Krista L. Lanctôt, Brian J. Murray, Ashley Cohen, Kevin E. Thorpe, Michelle N. Sicard, Karen Lien, Demetrios J. Sahlas, Nathan Herrmann

**Affiliations:** 1 University of Toronto, Toronto, Ontario, Canada; 2 Department of Medicine (Neurology), Sunnybrook Health Sciences Centre, Toronto, Ontario, Canada; 3 Hurvitz Brain Sciences Research Program, Toronto, Ontario, Canada; 4 Heart and Stroke Foundation Canadian Partnership for Stroke Recovery, Toronto, Ontario, Canada; 5 University of Toronto Stroke Program, Toronto, Ontario, Canada; 6 Department of Psychiatry, Sunnybrook Health Sciences Centre, Toronto, Ontario, Canada; 7 St. Michael’s Hospital, Applied Health Research Centre of the Li Ka Shing Knowledge Institute, Toronto, Ontario, Canada; 8 Dalla Lana School of Public Health, Toronto, Ontario, Canada; 9 McMaster University, Hamilton, Ontario, Canada; 10 Department of Medicine (Neurology), Hamilton Health Sciences, Hamilton, Ontario, Canada; 11 Hamilton General Hospital, Toronto, Ontario, Canada; University of Rome Tor Vergata, ITALY

## Abstract

**Background:**

Post-stroke Depression, Obstructive sleep apnea (OSA) and Cognitive impairment (“DOC”) are associated with greater mortality, worse recovery and poorer quality of life. Best practice recommendations endorse routine screening for each condition; yet, all are under-assessed, diagnosed and treated. We seek to determine the feasibility and validity of an integrated tool (“DOC” screen) to identify stroke clinic patients at high-risk of depression, OSA, and cognitive impairment.

**Methods:**

All consecutive new referrals to a regional Stroke Prevention Clinic who were English-speaking and non-aphasic were eligible to be screened. Time for screen completion was logged. DOC screen results were compared to the neuropsychological battery and polysomnogram assessments using a modified receiver operator characteristic and area under the curve analysis. Data is reported to conform to STARD guidelines.

**Findings:**

1503 people were screened over 2 years. 89% of eligible patients completed the screen in 5 minutes or less (mean 4.2 minutes), less than half the time it takes to complete the Montreal Cognitive Assessment (MoCA). 437 people consented to detailed testing. Of those, 421 completed the Structured Clinical Interview for Depression within 3 months of screening, 387 completed detailed neuropsychological testing within 3 months, and 88 had overnight polysomnograms. Screening scores combined with demographic variables (age, sex, education, body mass index), had excellent validity compared to gold standard diagnoses: DOC-Mood AUC 0.90; DOC-Apnea AUC 0.80; DOC-Cog AUC 0.81. DOC screen scores can reliably categorize patients in to low-, intermediate- or high-risk groups for further action and can do so with comparable accuracy to more time-consuming screens.

**Conclusions:**

Systematic screening of depression, obstructive sleep apnea, and cognitive impairment in 5 minutes or less is feasible and valid in a high volume stroke clinic using the DOC screen. The DOC screen may facilitate improved identification and treatment of these comorbidities to improve function in patients after stroke and in those with other neurological diseases that share these comorbid conditions (e.g. Alzheimer’s disease/mild cognitive impairment, Parkinson’s disease, Traumatic Brain Injury, multiple sclerosis).

## Introduction

The toll of stroke results from more than just brain injury. It is compounded by three common comorbidities: Depression, Obstructive sleep apnea (OSA), and Cognitive impairment (DOC), each affecting 30–50% of stroke clinic patients [1–5] All three impede recovery, are associated with poorer functional outcomes, worsen quality of life and increase the risk of recurrent stroke and mortality. [6] Best practice recommendations endorse routine screening [7–9] and screening tools for each condition abound. Yet, all are under-assessed, under-diagnosed, and under-treated in stroke clinic patients. [6] There are many reasons for this practice gap. [6] Best practice recommendations continue to endorse screening [7–9] and treatment [7–10] of severe symptoms for the immediate safety (e.g. driving assessments when moderate-to-severe cognitive impairment is identified) and quality of life (e.g. severe depression treatment) benefits. Despite these potential benefits, there remain challenges due to the high volume of stroke clinic patients, the time required for screening, potential confusion associated with multiple screening tools with varying degrees of evidence for their use, and the difficulties inherent in screening for complex outcomes like mood, sleep apnea and cognition. [6]

Based on the National Institute of Neurological Disorders and Stroke-Canadian Stroke Network (NINDS–CSN) panel recommendations, a 5-minute cognitive screen would be short enough for broad application in stroke prevention clinics and could facilitate screening across the spectrum of stroke care. [11] The Montreal Cognitive Assessment (MoCA) is a 30 point test that measures multiple cognitive domains, can be administered in roughly 10 minutes, and is sensitive to cognitive changes from stroke. [12, 13] However, given that many clinics see thousands of new patients per year, 10 minutes to obtain data on a single comorbidity is still too onerous for routine use. The low rates of screening (~10% of all new stroke prevention clinic (SPC) visits in an Ontario audit),[14] reflect this lack of routine uptake.

Multiple screens are available for use to detect depression, OSA and cognitive impairment. Studies evaluating the diagnostic characteristics of commonly used screens in different medical populations for these conditions report variable cut-points, sensitivities and specificities (S1 Appendix). The combined time to implement these screens is prohibitive for routine use. Prospective validation of brief, depression, OSA and cognitive screening tools that display robust psychometric properties are needed in the stroke population.

**Fig 1:**
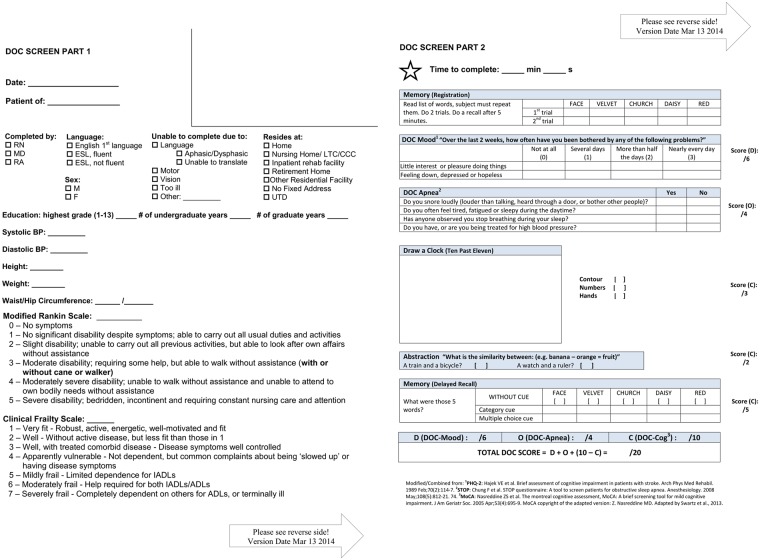
DOC screen.

We seek to determine whether a simple, evidence-based, integrated screening tool (which combines the PHQ-2, STOP questionnaire and a 10-point version of the MoCA) to identify individuals at high-risk of Depression, OSA, and Cognitive impairment, the “DOC” screen (Fig 1), is feasible and can reliably assess all three DOC conditions in a large-volume stroke prevention and TIA clinic.

## Methods

This study is designed and reported to conform to STARD guidelines for reporting studies of diagnostic accuracy.[15] Consecutive patients were screened for each comorbidity and consenting volunteers subsequently underwent the neuropsychological battery (NTP) and polysomnogram (PSG). A brief, integrated screening tool (Fig 1, www.docscreen.ca) was created using existing validated brief screens. The PHQ-2 (DOC-Mood), is a rapid screen for depression with two questions, scored from 0–3 (total 0–6) with established validity outside of stroke populations. [16–20] The STOP questionnaire (DOC-Apnea), is a four-question screen for OSA (scored 0–4) [21] which can be further modified with commonly available clinical data (BMI, Age, Neck circumference, sex (gender)–STOP-BANG), to increase sensitivity. [21] A previous study retrospectively extracted 10-points of the MoCA (5-word recall (5), clock drawing (3), and abstraction (2) and demonstrated strong predictive value for detecting cognitive impairment.[22] We selected this 10-point version of the MoCA (DOC-Cog) to prospectively validate in the stroke clinic population. We integrated the PHQ-2 (DOC-Mood) and STOP questionnaire (DOC-Apnea) into the delay between registration and recall of the 5 word recall task. This provides sufficient delay and distraction to maintain construct validity of the recall task. We collected data on routinely available demographic variables (age, sex, body mass index (BMI), years of education) to explore as covariates that might improve the diagnostic validity of the screening questionnaires.

Between April 23rd, 2012 and April 30th 2014, all consecutive new referrals to the regional SPC, who were English-speaking, not severely aphasic, and could see and write well enough to complete the screen, were assessed for inclusion. Data collection was planned before the index test and reference standard were performed. Screens were performed clinically for all patients; however, only those who could complete the test independently were included in feasibility analysis. The aim of the present study was to examine the relationship between a given stroke prevention clinic patient’s screen response and their detailed assessment scores. Patients with stroke, TIA and non-stroke diagnoses were included in the analyses as we were interested in the relevance of DOC screen results for guiding management across the broad spectrum of patients referred to the SPC. In addition, including patients without a stroke/TIA diagnosis improves the external validity of the screen as it will reflect the range of patients and performance seen across SPCs. A subset of these patients volunteered for the detailed testing. Mood was assessed using the Structured Clinical Interview for DSM Disorders (SCID-D) [23] as the gold standard for depression. Those classified as either minor or major depression by the SCID-D were considered to have depression. Minor depression was included in this definition given demonstrated impact on functional outcome [24–26] and recovery. [27] PSG was the gold standard assessment for OSA. Moderate-severe OSA was defined as an Apnea-Hypopnea Index (AHI) of ≥15, based on previous screening studies (S1 Appendix). The NTP was based on the 30-minute battery recommended by the NINDS-CSN harmonization paper,[11] which includes the Controlled Oral World Association Test (COWAT) of phonemic fluency, [28] Animal Naming task evaluating semantic fluency,[29] the California Verbal Learning Test (CLVT), [30] Digit Symbol Coding [31] and Trails Making A and B.[32] All scores were normalized for age, sex and education using data or z-scores from each respective test manual. Moderate-severe impairment was defined as 2 or more standard deviations (SD) from the mean score on 2 or more sub-tests of the battery, and was chosen to reflect severe impairment unlikely to be found by chance. In addition, patients completed an alternative version of the full MoCA.[12] This was not included in the definition of impairment. The Research Ethics Board of Sunnybrook Health Sciences Centre approved the protocol. Screening for these conditions is recommended by national best practice recommendations [7–9] so routine implementation was approved for waiver of consent to be screened and to track screening rates and times. Participants in the validation battery gave written informed consent. Stopwatches were used to record times. Clinical team members administered the screen according to instructions on the page (Fig 1).

Approximately 850 new outpatients are seen annually. Roughly 15% of patients in our clinic have aphasia or are non-English speaking. We estimated 720 patients would be screened annually. We conservatively estimated a 1/3 consent rate for gold standard testing, thus planned to test 240 patients annually. Half those who volunteered for the NTP were expected to undergo PSG, resulting in an estimated 240 sleep studies over 2 years.

The primary outcome measure was to determine whether the DOC screen was feasible. We defined feasibility as 85% of eligible patients completing the DOC screen in 5 minutes or less. The secondary outcome measures were to determine the levels of agreement between the DOC-Mood sub-score and a diagnosis of depression (minor or major classification on the SCID-D), DOC-Apnea sub-score and a diagnosis of moderate-severe OSA (AHI ≥ 15), and the DOC-Cog sub-score and the MoCA to impairment on the NTP (≥ 2SD from the mean on 2 or more subtests).

Statistical analysis was performed with R Version 3.0.3 (R Foundation for Statistical Computing) and SPSS version 22.0 software (SPSS Inc.). Descriptive statistics, including mean values and standard deviations were reported for age and number of years of education. The mean screen completion time and the percentage of patients who completed the screen in 5 minutes or less were calculated. Time to complete the DOC screen and the MoCA were compared using a paired samples t-test. One-way Analysis of Variance (ANOVA) was used to compare completion times across diagnoses. Significance was set at *p* < 0.05 for all analyses. Level of agreement between the screens and gold standard assessments was evaluated using receiver operating characteristic (ROC) and area under the curve analyses (AUC). Diagnostic cut-points were determined using a previously validated method. [33] Two diagnostic cut-points were determined using the ROC curve, and a cut-point with high sensitivity and a second cut-point with high specificity were determined. This analysis was run for each sub-score of the DOC screen. Positive predictive value (PPV), negative predictive value (NPV), Positive likelihood ratio (+LR) and Negative likelihood ratio (-LR) were also calculated. A logistic regression using clinically relevant variables such as age, sex, BMI and level of education was applied to the ROC curves for the DOC sub-scores, to further control for these factors when predicting impairment. No incomplete or indeterminate index or reference tests were included in the analysis.

## Results

### Population characteristics

**Fig 2:**
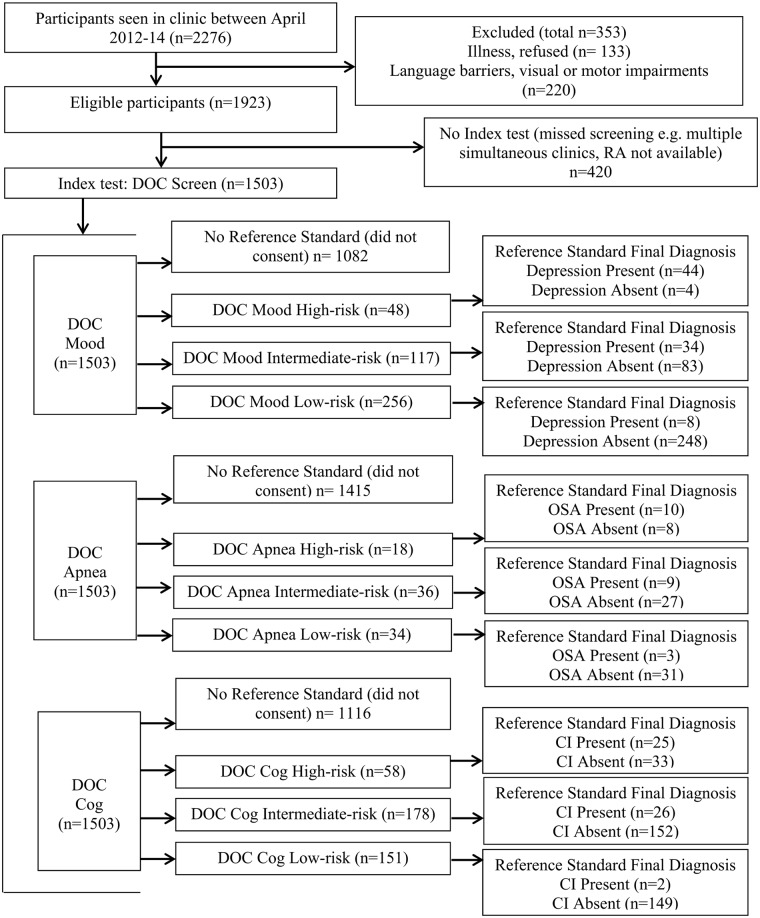
DOC study patient flow. Impairment on the reference standard was determined using the two cut-point regression method.

Patient flow through the study is summarized in Fig 2. During this period 2276 new referrals were identified. 420 were not approached or were missed (e.g. simultaneous patients seeing different physicians with only one research associate). Screens were attempted clinically for 353 patients who were identified as non-English speaking, aphasic, possessing motor/visual impairments, or whose illness would interfere with neuropsychological testing, however these patients were not included in the study sample based on a priori exclusion criteria. A total of 1503 eligible patients were screened and approached to complete detailed testing. 437 patients who were screened and included in the feasibility analysis gave informed consent to undergo more detailed testing. No significant differences were found in sex (x2 = 1.85, p = 0.174) or number of years of education (F(1,1504) = 0.26, p = 0.608) between included patients and missed patients, however missed patients were slightly younger (F(1,1921) = 4.54, p = 0.033).

Of the 1503 patients included in the feasibility analysis, 53% were female (Table 1). Mean age was 63.9±16.8 years (range: 16–100). Mean years of education was 14.7±3.9 years (range: 0–36). 558 (37%) participants had a stroke, 436 (29%) had a TIA, and 509 (34%) had an event other than stroke or TIA. Patients were commonly referred to the clinic for vascular risk reduction, asymptomatic carotid stenosis, white matter disease or transient neurological symptoms that were deemed to have other causes (e.g. stroke mimics such as migraine, seizure, benign paroxysmal positional vertigo). The 437 patients who volunteered to complete the NTP and PSG were slightly younger 62.7±15.6, and more highly educated 15.6±3.9. 155 participants (35%) had a stroke, 142 (33%) had a TIA and 140 (32%) were diagnosed with a condition other than stroke or TIA (Table 1).

**Table 1:** Feasibility and validity patient characteristics.

Feasibility	All patients (N = 2276)	Included patients (N = 1503)	Apriori excluded patients with completed DOC^a^ (N = 220)	Apriori excluded patients with incomplete DOC (N = 133)	Missed patients (N = 420)
Years of Education (N = 1776)	14.2±4.2 (0–36)	14.7±3.9 (0–36)	11.2±4.9 (1–25)	12±5 (0–28)	15.4±7 (6–25)
Age (N = 2276)	65.7±16.8 (15–100)	63.9±16.8 (16–100)	72.21±5.1 (21–98)	74.4±17.1 (18–96)	66.0±15.9 (15–96)
Sex (N = 2276)					
Male	1105 (49%)	1105 (49%)	1105 (49%)	1105 (49%)	1105 (49%)
Female	1171 (51%)	1171 (51%)	1171 (51%)	1171 (51%)	1171 (51%)
Event Type (N = 1503)					
Stroke	N/A	558 (37%)	N/A	N/A	N/A
TIA	N/A	436 (29%)	N/A	N/A	N/A
Other	N/A	509 (34%)	N/A	N/A	N/A
Validity	All (N = 437)	Complete Cog (N = 387)	Complete Mood (N = 421)	Complete PSG (N = 88)	Complete MoCA (N = 415)
Years of Education	15.6±3.9 (4–36)	15.7±3.8 (5–36)	15.2±3.9 (4–36)	15.4±3.8 (4–25)	15.6±3.8 (4–36)
Age	62.7±15.6 (17–95)	62.5±15.4 (17–94)	62.9±15.6 (17–95)	60.2±15.6 (17–91)	62.7±15.5 (17–94)
Sex					
Male	213 (49%)	184 (47%)	203 (48%)	35 (40%)	201(52%)
Female	224 (51%)	203 (53%)	218 (52%)	53 (60%)	214 (48%)
Event Type					
Stroke	155 (35%)	132 (34%)	148 (35%)	33 (38%)	145 (36%)
TIA	142 (33%)	130 (34%)	137 (33%)	30 (34%)	135 (32%)
Other	140 (32%)	125 (32%)	136 (32%)	25 (28%)	135 (32%)

^a^Apriori exclusions include non-English speaking patients, patients with motor/visual impairments or whose illness would interfere with neuropsychological testing

### Feasibility analyses

89% [87.7, 90.8] of eligible patients completed the screen in 5 minutes or less (mean 4.2 minutes [4.1, 4.3], range: 1.6–15.8 minutes, Table 2). Patients who consented to complete the PSG and NTP took less time to complete the screen than those who were screened without completing the NTP (Table 2). There was a statistically significant difference in time to complete between patients with stroke, TIA and other determined by one-way ANOVA (F(2,1502) = 12.154, p < .0001). A Tukey post-hoc test revealed that the time to complete the screen was significantly higher in stroke patients (4.4±1.6 minutes, p = .000) compared to TIA patients (4±1.5 minutes) as well as in stroke patients compared to patients who had an event other than stroke or TIA (4±1.4 minutes, p = .000). A comparative feasibility analysis was performed in the subset of patients who had consented to complete the NTP and had their time to complete the MoCA recorded (n = 286), and is summarized in Table 2. In comparison, only 8% [4.9, 11.5] of patients completed the MoCA in 5 minutes or less and the MoCA took significantly longer to complete than the DOC screen (mean 8.6 versus 4.2 minutes, p < .0001). The time to complete the MoCA ranged from 4.2–18.6 minutes and mean completion time was 8.6 minutes [8.3, 8.8].

**Table 2:** DOC screen and MoCA feasibility.

DOC Screen	Included patients (English, non-aphasic)	Apriori excluded patients with completed DOC N = 220^a^	Validity patients	Validity patients with completed MoCA
n	1503	220	437	286
Mean Time (minutes) [C.I]	4.2 [4.1, 4.3]	6.9 [6.5, 7.3]	3.8 [3.7, 3.9]	8.6 [8.3, 8.8]
Range, min-max (minutes)	1.6–15.8	1.6–15.8	2–9.6	4.2–18.6
Std. Deviation (minutes)	1.5	2.6	1.3	2.3
Completed in ≤ 5 min [C.I]	89% [87.7, 90.8]	41% [34.6, 48.2]	93% [91.1, 96]	8% [4.9, 11.5]

^a^Apriori exclusions include non-English speaking patients, patients with motor/visual impairments or whose illness would interfere with neuropsychological testing

### Validity analyses

The average time interval between index test and reference standard administration was 3 days. All PSG assessments were conducted within a year of screening. 421 patients completed the SCID-D, and 86 (20%) patients had minor or major depression according to the SCID-D, while 335 patients (80%) had no depression. 88 patients completed the overnight PSG. 22 (25%) patients were determined to have moderate-severe OSA according to the PSG, and in 67 patients (75%) no moderate-severe apnea was detected. 387 patients completed neuropsychological testing based on the 30-minute battery recommended by the NINDS-CSN harmonization paper. [11] 53 (14%) patients were determined to have moderate-severe cognitive impairment according to the NTP and 334 patients (86%) were found to have no severe impairment. Raw score data tables for DOC-Mood, DOC-Apnea, DOC-Cog and the MoCA by impairment on their respective gold standard assessments are found in Tables 3–6.

**Table 3:** DOC-Mood scores to impairment on the SCID-D.

DOC Mood Score	Not Impaired	Impaired
0	241	7
1	54	13
2	24	11
3	13	17
4	0	11
5	1	9
6	3	18

**Table 4:** DOC-Apnea scores to impairment on the polysomnogram.

DOC Apnea Score	Not Impaired	Impaired
0	6	1
1	17	3
2	20	5
3	20	8
4	3	5

**Table 5:** DOC-Cog scores to impairment on the neuropsychological test battery.

DOC Cog Score	Not Impaired	Impaired
0–3	0	0
4	3	4
5	13	8
6	17	7
7	33	9
8	70	16
9	93	9
10	105	0

**Table 6:** MoCA scores to impairment on the NTP.

MoCA Score	Not Impaired	Impaired
14	0	0
15	0	2
16	0	0
17	3	2
18	0	3
19	6	3
20	7	4
21	5	8
22	13	10
23	29	3
24	38	5
25	59	5
26	45	3
27	50	3
28	37	0
29	27	2
30	18	0

**Fig 3:**
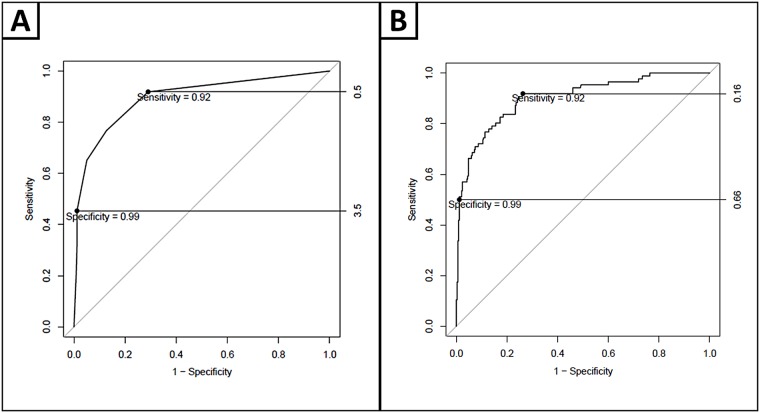
ROC curves displaying DOC mood optimal diagnostic cut-points, for high sensitivity and specificity and diagnostic cut-points with a logistic regression applied, controlling for age, sex and education.

Using the two-cut point approach,[33] AUC for the DOC-Mood is 0.898, sensitivity is optimal with DOC-Mood = 0 (sensitivity = 92%, NPV = 97%, -LR = 3.3), and specificity is optimal with DOC-Mood≥4 (specificity = 99%, PPV = 90%, +LR = 37) (Fig 3A). 10% of participants scored ≥4, and were classified as high-risk for depression. 59% participants scored 0, and these participants were classified as low-risk for depression. 31% of participants scored 1–3, and were classified as intermediate-risk for depression. When a logistic regression was applied to the ROC curve analysis (Fig 3B) controlling for age, sex and education, AUC = 0.902, sensitivity and specificity remained high (sensitivity = 92%, specificity = 99%). NPV remained high and -LR improved for the sensitive cut-point (97%, 3.5). PPV and +LR for the specific cut-point (92%, 42.9) is strengthened (Table 7). In addition, a smaller proportion (28%) of the population scored intermediate-risk. 29% of participants categorized as intermediate-risk are impaired according to the gold standard assessment.

**Table 7:** DOC-Mood results.

Diagnostic Characteristics	Single cut-point (raw scores)	Single cut-point (regression)	Two cut-points (raw scores)	Two cut-points (regression)
AUC	0.898	0.902	0.898	0.902
High-risk	Raw score 2–6	POI ^e^ > .201	Raw score 4–6	POI ^e^ > .62
% of population	106 (25%)	130 (31%)	42 (10%)	48 (11%)
Impaired	66 (62%)	71 (55%)	38 (90%)	44 (92%)
Not Impaired	40 (38%)	59 (45%)	4 (10%)	4 (8%)
Specificity	88%	82%	99%	99%
+LR ^a^	6.4	4.7	37	42.9
PPV ^b^	62%	55%	90%	92%
Intermediate-risk	N/A ^f^	N/A ^f^	Raw score 1–3	POI ^e^ > .16-.62
% of population	N/A ^f^	N/A ^f^	131 (31%)	117 (28%)
Impaired	N/A ^f^	N/A ^f^	41 (31%)	34 (29%)
Not Impaired	N/A ^f^	N/A ^f^	90 (69%)	83 (71%)
Specificity/Sensitivity	N/A ^f^	N/A ^f^	N/A ^g^	N/A ^g^
+/-LR ^a^^c^	N/A ^f^	N/A ^f^	N/A ^g^	N/A ^g^
PPV/NPV ^b^^d^	N/A ^f^	N/A ^f^	N/A ^g^	N/A ^g^
Low-risk	Raw score 0–1	POI ^e^ < .201	Raw score 0	POI ^e^ < .16
% of population	315 (75%)	291 (69%)	248 (59%)	256 (61%)
Impaired	20 (6%)	15 (5%)	7 (3%)	8 (3%)
Not Impaired	295 (93%)	276 (95%)	241 (97%)	248 (97%)
Sensitivity	77%	83%	92%	92%
-LR ^c^	0.3	0.2	3.3	3.5
NPV ^d^	94%	95%	97%	97%

^a^ +LR = positive likelihood ratio,

^b^ PPV = positive predictive value,

^c^ -LR = negative likelihood ratio,

^d^ NPV = negative predictive value,

^e^ POI = probability of impairment,

^f^ N/A, no intermediate group using single cut-point method,

^g^ cannot calculate statistics for intermediate group

**Fig 4:**
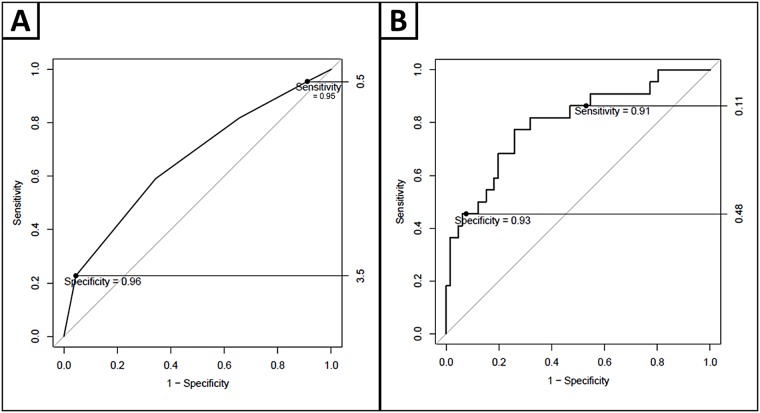
ROC curves displaying DOC apnea diagnostic cut-points, for high sensitivity and specificity and diagnostic cut-points with a logistic regression applied, controlling for age, sex and BMI.

For obstructive sleep apnea, AUC is 0.660, sensitivity is optimal with DOC-Apnea = 0 (sensitivity = 95%, NPV = 86%, -LR = 1.1), and specificity is optimal with DOC-Apnea = 4 (specificity = 96%, PPV = 63%, +LR = 5) (Fig 4A). 9% of participants scored 4, and were classified as high-risk for OSA. 8% participants scored 0, and these participants were classified as low-risk for OSA. 83% of participants scored 1–3, and were classified as intermediate-risk for OSA. When a logistic regression was applied to the ROC curve analysis controlling for age, sex and BMI (Fig 4B), AUC improved to 0.798, sensitivity and specificity remained high (sensitivity = 91%, specificity = 93%). NPV and -LR for the sensitive cut-point (91%, 1.6) are strengthened. PPV and +LR for the specific cut-point (56%, 3.8) are slightly weakened (Table 8). The proportion of the population who are categorized as intermediate-risk decreases considerably (41% versus 83%). 25% of participants categorized as intermediate-risk are impaired according to PSG.

**Table 8:** DOC-Apnea results.

Diagnostic Characteristics	Single cut-point (raw scores)	Single cut-point (regression)	Two cut-points (raw scores)	Two cut-points (regression)
AUC	0.660	0.798	0.660	0.798
High-risk	Raw score 3–4	POI ^e^ > .19	Raw score 4	POI ^e^ > .43
% of population	36 (41%)	40 (45%)	8 (9%)	18 (20%)
Impaired	13 (36%)	18 (45%)	5 (63%)	10 (55%)
Not Impaired	23 (64%)	22 (55%)	3 (37%)	8 (45%)
Specificity	65%	66%	96%	93%
+LR ^a^	1.7	2.5	5	3.8
PPV ^b^	36%	45%	63%	56%
Intermediate-risk	N/A ^f^	N/A ^f^	Raw score 1–3	POI ^e^ 0.11-.43
% of population	N/A ^f^	N/A ^f^	73 (83%)	36 (41%)
Impaired	N/A ^f^	N/A ^f^	16 (22%)	9 (25%)
Not Impaired	N/A ^f^	N/A ^f^	57 (78%)	27 (75%)
Specificity/Sensitivity	N/A ^f^	N/A ^f^	N/A ^g^	N/A ^g^
+/-LR ^a^^c^	N/A ^f^	N/A ^f^	N/A ^g^	N/A ^g^
PPV/NPV ^b^^d^	N/A ^f^	N/A ^f^	N/A ^g^	N/A ^g^
Low-risk	Raw score 0–2	POI ^e^ < .19	Raw score 0	POI ^e^ < .11
% of population	52 (59%)	48 (55%)	7 (8%)	34 (39%)
Impaired	9 (17%)	4 (17%)	1 (14%)	3 (12%)
Not Impaired	43 (83%)	44 (83%)	6 (86%)	31 (88%)
Sensitivity	59%	82%	95%	91%
-LR ^c^	0.6	0.3	1.1	1.6
NPV ^d^	83%	92%	86%	91%

^a^ +LR = positive likelihood ratio,

^b^ PPV = positive predictive value,

^c^ -LR = negative likelihood ratio,

^d^ NPV = negative predictive value,

^e^ POI = probability of impairment,

^f^ N/A, no intermediate group using single cut-point method,

^g^ cannot calculate statistics for intermediate group

**Fig 5:**
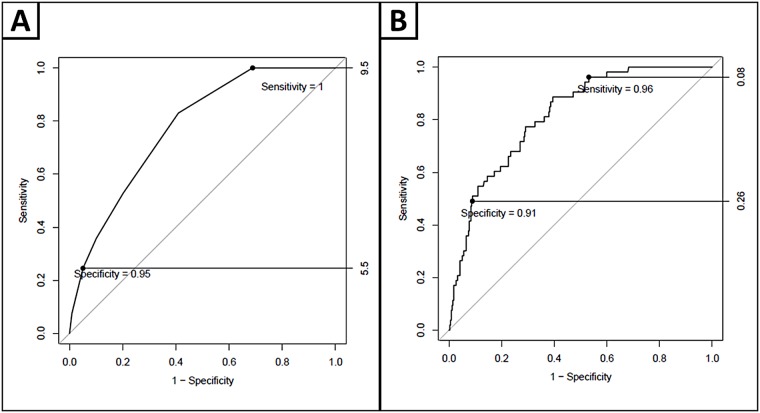
ROC curves displaying DOC cog diagnostic cut-points, for high sensitivity and specificity and with diagnostic cut-points with a logistic regression applied, controlling for age, sex and education.

For cognitive impairment, AUC of the DOC-Cog screening is 0.776; sensitivity is optimal with DOC-Cog = 10 (sensitivity = 100%, NPV = 100%, -LR = 1.5), and specificity is optimal with DOC-Cog≤ 5 (specificity = 95%, PPV = 43%, +LR = 4.7) (Fig 5A). 7% of participants scored ≤5, and were classified as high-risk for cognitive impairment. 27% participants scored 10, and these participants were classified as low-risk for cognitive impairment. 66% of participants scored 6–9, and were classified as intermediate-risk for cognitive impairment. When a logistic regression was applied to the ROC curve analysis (Fig 5B) controlling for age, sex and education, AUC is 0.814, sensitivity and specificity remained high (sensitivity = 96%, specificity = 91%). NPV remains high (99%) and -LR for the sensitive cut-point improves (1.7). PPV is unchanged (43%) and +LR for the specific cut-point (4.8) is strengthened (Table 9). The proportion of the population that can be categorized as intermediate-risk decreases (46% versus 66%). 15% of categorized as intermediate-risk are impaired according to the NTP.

**Table 9:** DOC-Cog results.

Diagnostic Characteristics	Single cut-point (raw scores)	Single cut-point (regression)	Two cut-points (raw scores)	Two cut-points (regression)
AUC	0.776	0.814	0.776	0.814
High-risk	Raw score 0–8	POI ^e^ > .122	Raw score 0–5	POI ^e^ > .26
% of population	180 (47%)	180 (47%)	28 (7%)	58 (15%)
Impaired	44 (24%)	47 (26%)	12 (41%)	25(42%)
Not Impaired	136 (76%)	133 (74%)	16 (59%)	33 (58%)
Specificity	59%	61%	95%	91%
+LR ^a^	2	2.2	4.7	4.8
PPV ^b^	24%	26%	43%	43%
Intermediate-risk	N/A ^f^	N/A ^f^	Raw score 6–9	POI ^e^ .08-.26
% of population	N/A ^f^	N/A ^f^	254 (66%)	178 (46%)
Impaired	N/A ^f^	N/A ^f^	41 (16%)	26 (15%)
Not Impaired	N/A ^f^	N/A ^f^	213 (84%)	152 (85%)
Specificity/Sensitivity	N/A ^f^	N/A ^f^	N/A ^g^	N/A ^g^
+/-LR ^a^^c^	N/A ^f^	N/A ^f^	N/A ^g^	N/A ^g^
PPV/NPV ^b^^d^	N/A ^f^	N/A ^f^	N/A ^g^	N/A ^g^
Low-risk	Raw score 9–10	POI ^e^ < .122	Raw score 10	POI ^e^ < .08
% of population	207 (53%)	207 (53%)	105 (27%)	151 (39%)
Impaired	9 (4%)	6 (3%)	0 (0%)	2 (1%)
Not Impaired	198 (96%)	201 (97%)	105 (100%)	149 (99%)
Sensitivity	83%	88%	100%	96%
-LR ^c^	0.3	0.2	1.5	1.7
NPV ^d^	96%	97%	100%	99%

^a^ +LR = positive likelihood ratio,

^b^ PPV = positive predictive value,

^c^ -LR = negative likelihood ratio,

^d^ NPV = negative predictive value,

^e^ POI = probability of impairment,

^f^ N/A, no intermediate group using single cut-point method,

^g^ cannot calculate statistics for intermediate group

## Discussion

The DOC screen is an integrated tool to assess risk for depression, OSA and cognitive impairment that can be feasibly applied in 5 minutes or less, in a large-volume clinic. Patients not fluent in English or with aphasia were excluded from the feasibility analysis; many completed the screen, but required more time. Using the DOC screen, clinically relevant information can be obtained for all three important post-stroke comorbidities, in less than half the time required to complete only the MoCA. The DOC screen, while efficient, is also not too short. The overall mean time of 4.2 minutes is long enough to maintain the validity of the delayed recall task.

The DOC screen was validated as a composite tool, using the multiple ROC curve cut-point methodology published previously.[33] Our results display excellent diagnostic characteristics for the PHQ-2, STOP and MoCA components (Tables 7–9). DOC-Mood displayed excellent sensitivity and specificity for detecting depression using the two cut-point approach (92%, 99%). Of each mini-screen, the DOC-Mood exhibited the most robust diagnostic characteristics and had the lowest percentage of participants (28%) scoring in the intermediate-risk, after controlling for clinically significant variables. 29% of patients who scored intermediate-risk were impaired according to the SCID-D. Therefore, clinicians may use caution when a patient at intermediate-risk for depression is identified by applying a more detailed screening tool, or pairing the DOC-Mood with additional clinical questions.

DOC-Apnea displayed strong sensitivity and specificity for detecting OSA with the two-cut-point approach (95%, 96%); however, too many patients (81%) scored intermediate-risk. A very small percentage of patients who were included in the study agreed to undergo PSG. Given the challenge of recruiting patients for PSGs, it was necessary to include all patients who had PSGs within a year (+/-) of screening. This may limit our results because by the time patients underwent their PSG, post-stroke apnea symptoms identified at screening may have improved or even resolved. Conversely, patients who underwent sleep studies prior to screening where moderate-severe OSA was identified may have undergone treatment, reducing sleep apnea symptoms at the time of screening. Despite these limitations, controlling for clinically significant variables significantly reduced the proportion of patients who can be categorized as intermediate-risk for OSA (81% versus 41%), while maintaining high sensitivity and specificity. Variables for the logistic regression model were based on the STOP-BANG questionnaire (age, sex, BMI, with the exception of neck circumference, as it is not routinely collected clinically and short administration time was paramount).[21] Controlling for age, sex and BMI (“STOP-BAG”) adds significant predictive value for the sensitive cut-point, but not the specific cut-point, suggesting that these variables enhance the screening tool’s ability to rule out sleep apnea. Paper screens for OSA are simple but have limited ability to detect OSA for many stroke clinic patients. Other screening methods such as home monitoring may also be a viable option but require further study.

DOC-Cog displays excellent sensitivity and specificity (100%, 95%) for detecting cognitive impairment using the two cut-point approach. The +LR is strong for the specific cut-point, however PPV is relatively low, suggesting that the DOC-Cog is more reliable to rule out moderate-severe impairment than for ruling it in. This relationship was also observed using the full MoCA.[33] DOC-Cog logistic regression modeling controlling for age, sex and education reduced the number of participants who score intermediate-risk from 66% to 46%, while maintaining excellent sensitivity and specificity. The DOC-Cog is an accurate alternative tool compared to the MoCA for detecting cognitive impairment in busy clinic settings.

We were interested in the relevance of DOC screen results for guiding management across the broad spectrum of patients referred to the SPC. The DOC conditions may be relevant for guiding management in not only stroke patients, but TIA patients and common stroke mimics as well, and so all eligible stroke clinic patients were included in the analyses. The purpose of the study was to examine the relationship between a given stroke prevention clinic patient’s screen response and their detailed assessment scores. Since there is a wide range of performance seen within our population, including patients without a stroke/TIA diagnosis does not significantly limit the validation. Additionally, inclusion of these patients may improve the external validity of the screen as it will reflect the range of patients and performance seen across SPCs.

Our study was rigorously conducted in a large sample size and reported to conform to STARD guidelines, but there remain several important limitations. Conceptually it is vital to recognize that the screen does not measure a person’s day-to-day function, assess for change from baseline, or quantify the duration, triggers, or acuity of symptoms. Intermediate or high screen scores should prompt further inquiry. Additionally, scores for one condition may affect the others. For example, patients with depression may frequently score higher on apnea questionnaires due to fatigue, but this may be due to depression, rather than risk of OSA, and vice versa. There are also limitations to each screen sub-component. The depression sub-screen questions are framed for the two-week period prior to the patient’s first clinic visit. This reflects risk of a Major Depressive Episode, not a Major Depressive Disorder diagnosis and may be biased by their recent stroke event. The STOP questionnaire utilizes self-report questions that are difficult for patients without a bed partner (e.g. snoring, observed apneas). The brief cognitive screen covers the majority of the domains of the MoCA including frontal/executive dysfunction, but is heavily language based. Additionally, the screen is designed for outpatient clinic environments. More detailed assessments (PHQ-9, PSG, portable apnea monitoring, MoCA) are likely appropriate for acute care or rehabilitation hospital inpatients who are assessed for a longer period of time. Screening tools designed for outpatients with aphasia, motor or sight impairments must be used as appropriate as their exclusion from this study may have underestimated the frequency and severity of post-stroke DOC. Other limitations are associated with the study sample. The very low uptake of PSG likely reflects real-world challenges in the assessment and treatment of OSA. Further, those patients that volunteered for the study were milder than the total population screened—they tended to complete the screen faster and had slightly lower rates of high-risk responses to screening components. Most validation studies do not quantify the number and characteristics of patients who decline participation in research studies, and in this regard, our study is more rigorous. The “healthy volunteer” bias is common but this effect was mild in our cohort. Since the purpose was to examine the relationship between a given person’s screen response and their detailed assessment scores, and since there is a wide range of performance seen within our population, this “healthy volunteer bias” does not significantly limit the validation. While our sample size is large, this study took place at a single centre as study funding did not permit a multi-centre design. However, validation studies of similar tests (e.g. MoCA) often start with single site designs, and we hope that future research will replicate our findings. Finally, the research assistants performing the screening or reference test were not always blinded to the other results. In some cases, the same person may have administered both tests, due to limited research personnel availability.

Screening for the DOC conditions remains limited in stroke clinics. The most significant reason for this gap is a lack of evidence for effective treatments of each condition from randomized controlled trials in stroke patients.[6] Notwithstanding this controversy, there are several important reasons to screen, including symptom reduction, safety concerns, quality of life improvements and practical concerns such as medico-legal issues.[6] Screening should not focus on a definite “yes-no” diagnosis, but rather should form the basis for a pragmatic approach to navigating clinical care pathways and research selection. Our approach creates useful clinical categories—those of low-level concern who are very unlikely to have DOC conditions and thus do not require immediate management, those of intermediate-level concern with possible presence of DOC comorbidities, who should be monitored or further assessed, and those with greatest concern, for whom management, intervention, or appropriate follow-up is necessary. To ensure continuity of treatment based on screening results, structured procedures for referrals to health care professionals specializing in management of the DOC conditions must be established, but may differ between institutions. In order to minimize the number of patients in the intermediate-risk category for which follow-up actions may be unclear, we have developed a regression model using DOC scores and demographic and clinical information (age, sex, BMI, education) which is publicly available for use at www.docscreen.ca. Controlling for these variables leads to less patients in the intermediate category and greater predictive value of the DOC screen. The DOC screen’s brevity permits broad screening for these important health conditions. Best practice recommendations endorse routine screening for each condition in stroke clinic patients, [7–9] yet all three are routinely under assessed and under treated. [6] Efficient screening will facilitate early identification and assessment of patients at highest-risk of each disease.

## Conclusion

The DOC screen is a feasible and valid tool that can reliably identify stroke clinic patients at high-risk of depression, OSA and cognitive impairment in minutes 5 or less in high volume stroke clinics. Given that these conditions are also highly prevalent in other neurological and vascular disorders (e.g. Multiple Sclerosis, Alzheimer’s disease/mild cognitive impairment, congestive heart failure) these data may be of significant interest to the broader medicine audience. **The DOC screen is publicly available for download at** www.docscreen.ca**. Reports of the risk category for each DOC screen component can be generated freely at** www.docscreen.ca **using either raw scores or regression-based approaches (the regression-based approach has fewer people categorized as intermediate-risk).**

## Supporting information

S1 Appendix Supplementary Table I: Diagnostic characteristics of commonly used brief screening tests for DOC conditions. (DOCX)

S1 Dataset DOC validity dataset. (XLSX)

S2 Dataset DOC feasibility dataset. (XLSX)
