# Supplementary material for: The “DOC” screen: Feasible and valid screening for depression, Obstructive Sleep Apnea (OSA) and cognitive impairment in stroke prevention clinics
Source: PLoS One. 2017 Apr 4;12(4):e0174451. doi: 10.1371/journal.pone.0174451 (PMC5380324; doi:10.1371/journal.pone.0174451)
Supplement: S1 Appendix — (DOCX) [file pone.0174451.s001.docx]

## Supplementary Table I: Diagnostic Characteristics of Commonly Used Brief Screening Tests for DOC Conditions

|  | **Optimal**  **Cut-point** | **Sensitivity (%)** | **Specificity (%)** |
| --- | --- | --- | --- |
| **Depression**  **Patient Health Questionnaire-9 ^a^**  Williams (2005)^1^  Prisnie (2016)^2^  **Patient Health Questionnaire-2**  ^b^ Arrol (2010)^3^  ^b^ Kroenke (2003)^4^  ^b^ Thombs (2008)^5^  ^b^ Lowe (2005)^6^  ^b^ Boyle (2011)^7^  ^a^ Prisnie (2016)^2^  **Hospital Anxiety and Depression Scale ^a^**  Johnson (1995)^8^  Sagen (2009)^9^  Aben (2002)^10^  Tang (2010)^11^  **Geriatric Depression Scale ^a^**  Johnson (1995)^8^  Agrell (1989)^12^  Tang (2004)^11^ | ≥ 10  ≥ 13  ≥ 2  ≥ 2  ≥ 3  ≥ 2  ≥ 3  ≥ 3  ≥ 4  ≥ 4  ≥ 7  ≥ 6  ≥ 10  ≥ 10  ≥ 6 | 91  82  86  83  82  79  78  75  83  84  73  88  84  88  89 | 89  97  78  92  79  86  71  96  44  73  79  53  66  64  73 |
| **OSA**  **Berlin Questionnaire**  ^a^ Elkholy (2012)^13^  ^a^ Kotzian (2012)^14^  ^a^ Srijithesh (2011)^15^  ^a^  Joo (2011)^16^  ^b^ Chung (2008a)^17, 18^  ^b^ Chung (2008a) ^17, 18^  ^b^ Netzer (1999)^18,19^  **Epworth Sleepiness Scale ^a^**  Camilo (2014)^20^  Joo (2011)^16^  Arzt (2010)^21^  **STOP ^b^**  Chung (2008a)^17^  Chung (2008a)^17^  **Modified STOP (BANG-BAG)**  ^b^ Chung (2008b) ^18, 22^  ^b^ Chung (2008b) ^18, 22^  ^a^ Boulos (2016)^23^ | ≥ 2 positive categories  ≥ 10  ≥ 10  ≥ 10  ≥ 2  ≥ 2  ≥ 2  ≥ 2  ≥ 2 | 57  69  77  Not reported  87  79  54  Not reported  Not reported  Not reported  79  74  100  93  94 | 86  15  54  Not reported  46  50  97  Not reported  Not reported  Not reported  49  43  37  43  14 |
| **Cognitive Impairment**  **Montreal Cognitive Assessment^a^**  Cumming (2013)^24^  Salvadori (2013)^25^  Dong (2010)^26^  Dong (2012)^27^  Lees (2013)^28, iii^  Godefroy (2011)^29,^ ^i^  Wong (2009)^30^  Godefroy (2011)^29,ii^  Wu (2013)^31^  **Mini-Mental State Examination^a^**  Bour (2010)^32^  Cumming (2013)^24^  Tang (2005)^33^  Dong (2012)^27^  Dong (2010)^26^  Desmond (1994)^34^  Bour (2010)^32^  Lees (2013)^28^, ^iii^  Godefroy (2011)^29,^ ^i^  Godefroy (2011)^29, ii^  Blake (2002)^35^  Agrell (200)^36^  Morris (2012)^37^  Srikanth (2006)^38^ | ≤23  ≤21  ≤21  ≤21  ≤21  ≤22  ≤21  ≤19  ≤23  ≤23  ≤26  ≤18  ≤25  ≤24  ≤24  ≤26  ≤24  ≤24  ≤24  ≤24  ≤23  ≤24  ≤23 | 92  91  90  88  84  78  73  69  65    96  94  91  88  86  83  82  71  70  70  62  56  55  14 | 67  76  77  64  78  90  75  94  79    83  75  88  67  82  87  75  85  97  94  88  24  60  100 |

All optimal cut-points are chosen for detecting minor-major depression, moderate-severe OSA and moderate-severe cognitive impairment or dementia.

^a^ Studies were conducted in stroke populations

^b^ Studies were not conducted in the stroke population

^i^ Optimal adjusted cut-off reported, ii Optimal raw score reported, ^iii^ Pooled sensitivity and specificity reported

# References

1. Williams, LS, Brizendine, EJ, Plue, L, Bakas, T, Tu, W, Hendrie, H, et al. Performance of the PHQ-9 as a screening tool for depression after stroke. Stroke 2005; 36: 635–638.

2. Prisnie JC, Fiest KM, Coutts SB, Patten SB, Atta CA, Blaikie L, et al. Validating screening tools for depression in stroke and transient ischemic attack patients. *Int J Psychiatry Med*. 2016;51:262-277.

3. Arroll, B, Goodyear-Smith, F, Crengle, S, Gunn, J, Kerse, N, Fishman, T, et al. Validation of PHQ-2 and PHQ-9 to screen for major depression in the primary care population. Ann Fam Med 2010;8:348–53.

4. Kroenke, K, Spitzer, RL, Williams, JB. The Patient Health Questionnaire-2: validity of a two-item depression screener. Med Care 2003;41:1284–1292.

5. Thombs, BD, Ziegelstein, RC, Whooley, MA. Optimizing detection of major depression among patients with coronary artery disease using the patient health questionnaire: data from the heart and soul study. J Gen Intern Med 2008;23:2014–2017.

6. Lowe, B, Kroenke, K, Grafe, K. Detecting and monitoring depression with a two-item questionnaire (PHQ-2). J Psychosom Res 2005;58:163–171.

7. Boyle, LL, Richardson, TM, He, H, Xia, Y, Tu, X, Boustani, M, et al. How do the phq-2, the phq-9 perform in aging services clients with cognitive impairment? Int J Geriatr Psychiatry 2011;26:952-960.

8. Johnson, G, Burvill, PW, Anderson, CS, Jamrozik, K, Stewart-Wynne, EG, Chakera, TM.. Screening instruments for depression and anxiety following stroke: experience in the Perth community stroke study. Acta Psychiatr Scand 1995;91:252–257.

9. Sagen, U, Vik, TG, Moum, T, Morland, T, Finset, A, Dammen, T, et al. Screening for anxiety and depression after stroke: comparison of the hospital anxiety and depression scale and the Montgomery and Asberg depression rating scale. J Psychosom Res 2009;67:325–32.

10. Aben, I, Verhey, F, Lousberg, R, Lodder, J, Honig, A. Validity of the beck depression inventory, hospital anxiety and depression scale, SCL-90, and hamilton depression rating scale as screening instruments for depression in stroke patients. Psychosomatics (2002;43: 386–393.

11. Tang, WK, Ungvari, GS, Chiu, HF, Sze, KH, Yu, AC, Leung, TL. Screening post-stroke depression in Chinese older adults using the hospital anxiety and depression scale. Aging Ment.Health 2010;8:397–399.

12. Agrell, B, Dehlin, O. Comparison of six depression rating scales in geriatric stroke patients. Stroke 1989;20:1190–1194.

13. Elkholy SH, Amer HA, Nada MM, Nada MAF, Labib A. Sleep-Related Breathing Disorders in Cerebrovascular Stroke and Transient Ischemic Attacks : A Comparative Study. J Clin Neurophysiol 2012;29:194-198.

14. Kotzian ST, Stanek JK, Pinter MM, Grossmann W, Saletu MT. Subjective Evaluation of Sleep Apnea Is Not Suffi cient in Stroke Rehabilitation. Top Stroke Rehabil 2012;19:45-53.

15. Srijithesh, PR, Shukla, G, Srivastav, A, Goyal, V, Singh, S, Behari, M. Validity of the Berlin Questionnaire in identifying obstructive sleep apnea syndrome when administered to the informants of stroke patients. J Clin Neurosci 2011;18:340–343.

16. Joo BE, Seok HY, Yu SW, Kim BJ, Park KW, Lee DH, et al. Prevalence of sleep-disordered breathing in acute ischemic stroke as determined using a portable sleep apnea monitoring device in Korean subjects. Sleep Breath 2011;15:77-82.

17. Chung, F, Yegneswaran, B, Liao, P, Chung, SA, Vairavanathan, S, Islam, S, et al. Validation of the Berlin questionnaire and American Society of Anesthesiologists checklist as screening tools for obstructive sleep apnea in surgical patients. Anesthesiology 2008;108:822–30.

18. Abrishami, A, Khajehdehi, A, Chung, F. A systematic review of screening questionnaires for obstructive sleep apnea. Can J Anaesth 2919;57:423–438.

19. Netzer, NC, Stoohs, RA, Netzer, CM, Clark, K, Strohl, KP. Using the Berlin Questionnaire to identify patients at risk for the sleep apnea syndrome. Ann Intern Med 1999;131:485–91.

20. Camilo MR, Sander HH, Eckeli AL, Fernandes RM, Dos Santos-Pontelli TE, Leite JP, et al. SOS score: an optimized score to screen acute stroke patients for obstructive sleep apnea. Sleep Med 2014;15:1021-1024.

21. Arzt M, Young T, Peppard PE, Finn L, Ryan CM, Bayley M, et al. Dissociation of obstructive sleep apnea from hypersomnolence and obesity in patients with stroke. Stroke 2010;41:e129-e134.

22. Chung, F, Yegneswaran, B, Liao, P, Chung, SA, Vairavanathan, S, Islam, S, et al. STOP questionnaire: a tool to screen patients for obstructive sleep apnea. Anesthesiology 2008;108:812–821.

23. Boulos MI, Wan A, Im J, Elias S, Frankul F, Atalla M, et al. Identifying obstructive sleep apnea after stroke / TIA : evaluating four simple screening tools. Sleep Med 2016;21:133-139.

24. Cumming TB, Churilov L, Linden T, Bernhardt J. Montreal cognitive assessment and mini-mental state examination are both valid cognitive tools in stroke. Acta Neurol Scand. 2013;128:122-129.

25. Salvadori E, Pasi M, Poggesi A, Chiti G, Inzitari D, Pantoni L. Predictive value of MoCA in the acute phase of stroke on the diagnosis of mid-term cognitive impairment. J Neurol 2013;260:2220-2227.

26. Dong Y, Sharma VK, Chan BP, Venketasubramanian N, Teoh HL, Seet RC, et al. The Montreal Cognitive Assessment (MoCA) is superior to the Mini-Mental State Examination (MMSE) for the detection of vascular cognitive impairment after acute stroke. J Neurol Sci 2010;299:15-8.

27. Dong Y, Venketasubramanian N, Chan BP, Sharma VK, Slavin MJ, Collinson SL, et al. Brief screening tests during acute admission in patients with mild stroke are predictive of vascular cognitive impairment 3-6 months after stroke. J Neurol Neurosurg Psychiatry 2012;83:580-585.

28. Lees R, Selvarajah J, Fenton C, Pendlebury ST, Langhorne P, Stott DJ, et al. Test Accuracy of Cognitive Screening Tests for Diagnosis of Dementia and Multidomain Cognitive Impairment in Stroke. Stroke 2014;45:3008-3018.

29. Godefroy O, Fickl A, Roussel M, Auribault C, Bugnicourt JM, Lamy C, et al. Is the montreal cognitive assessment superior to the mini-mental state examination to detect poststroke cognitive impairment?: A study with neuropsychological evaluation. Stroke 2011;42:1712-1716.

30. Wong A, Xiong YY, Kwan PWL, Chan AY, Lam WW, Wang K, et al. The validity, reliability and clinical utility of the Hong Kong Montreal Cognitive Assessment (HK-MoCA) in patients with cerebral small vessel disease. Dement Geriatr Cogn Disord 2009;28:81-87.

31. Wu Y, Wang M, Ren M, Xu W. The effects of educational background on Montreal Cognitive Assessment screening for vascular cognitive impairment, no dementia, caused by ischemic stroke. J Clin Neurosci 2013;20:1406-1410.

32. Bour, A, Rasquin, S, Boreas, A, Limburg, M, Verhey, F. How predictive is the MMSE for cognitive performance after stroke? J Neurol 2010;257:630–637.

33. Tang, WK, Mok, V, Chan, SS, Chiu, HF, Wong, KS, Kwok, TC, et al. Screening of dementia in stroke patients with lacunar infarcts: comparison of the mattis dementia rating scale and the mini-mental state examination. J Geriatr Psychiatry Neurol 2005;18:3–7.

34. Desmond, DW, Tatemichi, TK, Hanzawa, L. The Telephone Interview for Cognitive Status: Reliability and Validity in a Stroke Sample. Ann Neurol 1993;34:258–259.

35. Blake, H, McKinney, M, Treece, K, Lee, E, Lincoln, NB. An evaluation of screening measures for cognitive impairment after stroke. Age Ageing 2002;31:451–456.

36. Agrell, B, Dehlin, O. Mini Mental State Examination in geriatric stroke patients. Validity, differences between subgroups of patients, and relationships to somatic and mental variables. Aging 2000;12:439–444.

37. Morris, K, Hacker, V, Lincoln, NB. The validity of the Addenbrooke’s Cognitive Examination-Revised (ACE-R) in acute stroke. Disabil Rehabil 2012;34:189–95.

38. Srikanth, V, Thrift, AG, Fryer, JL, Saling, MM, Dewey, HM, Sturn, JW, et al. The validity of brief screening cognitive instruments in the diagnosis of cognitive impairment and dementia after first-ever stroke. Int Psychogeriatr 2006;18:295–305.
